# Supplementary material for: Direct CD32 T-cell cytotoxicity: implications for breast cancer prognosis and treatment
Source: Life Sci Alliance. 2022 Oct 14;5(12):e202201590. doi: 10.26508/lsa.202201590 (PMC9586128; doi:10.26508/lsa.202201590)
Supplement: Supplementary file 1 [file LSA-2022-01590_TableS1.docx]

**Table1S. List of cells screened for induction of CD32-CR downregulation in CD32A-T cells**

| Tissue Type | Cell Type |
| --- | --- |
| Breast | MDA-MB-468 |
|  | MDA-MB-231 |
|  | HCC-1954 |
|  | MCF7 |
|  | SKBR3 |
|  | SUM-159  T-47-D |
| Colon | HCT116 |
|  | HT29 |
| Head&Neck | A-253 |
|  | FaDu |
| Epithelial Lung (NSCLC) | A549 |
| Hematopoietic | ML-2 |
|  | U937 |
|  | Jurkat |
| Endothelium | HUVEC |
| Normal Lung Fibroblast | IMR-90 |
| Normal Skin Fibroblast | BJ |
| Normal Myoblast | h-MB |
